# Fast Noncircular 2D-DOA Estimation for Rectangular Planar Array

**DOI:** 10.3390/s17040840

**Published:** 2017-04-12

**Authors:** Lingyun Xu, Fangqing Wen

**Affiliations:** 1 College of Science, Nanjing University of Aeronautics & Astronautics, Nanjing 210016, China; 2 Department of Electrical and Computer Engineering, University of Delaware, Newark, DE 19716, USA; 3 Electronic and Information School of Yangtze University, Jingzhou 434023, China; wfqzhc@163.com

**Keywords:** two-dimensional direction of arrival (2D-DOA), real-valued propagator method, noncircular, rectangular planar array

## Abstract

A novel scheme is proposed for direction finding with uniform rectangular planar array. First, the characteristics of noncircular signals and Euler’s formula are exploited to construct a new real-valued rectangular array data. Then, the rotational invariance relations for real-valued signal space are depicted in a new way. Finally the real-valued propagator method is utilized to estimate the pairing two-dimensional direction of arrival (2D-DOA). The proposed algorithm provides better angle estimation performance and can discern more sources than the 2D propagator method. At the same time, it has very close angle estimation performance to the noncircular propagator method (NC-PM) with reduced computational complexity.

## 1. Introduction

Two-dimensional direction of arrival (2D-DOA) estimation has been widely used in mobile communication systems, sonar, navigation, radar, etc. [1,2,3,4,5], which is an important research branch in array signal processing. Many 2D-DOA estimation algorithms have sprung up in recent years in order to improve the performance of angle estimation, which include the two dimensional multiple signal classification(2D MUSIC) algorithm [6], the 2D Unitary estimation of signal parameters via rotational invariance techniques (ESPRIT) algorithm [7], the modified 2D-ESPRIT algorithm [8], the matrix pencil method [9], the maximum likelihood method [10,11], the parallel factor (PARAFAC) algorithm [12], and so on [13,14,15,16,17,18,19,20]. However, those 2D-DOA estimation algorithms are confronted with the problem of the high computational complexity generally and they are very difficult to apply in engineering practice. As is known to us, the propagator method (PM) algorithm uses linear operations to replace the eigenvalue decomposition of the covariance matrix [21], and it has a great advantage in resolving the amount of calculation. Therefore, the 2D-DOA estimation based on PM is becoming a hot spot of research. For example, Wu et al. have developed the 2D-DOA estimation algorithm via the rotational invariance property of propagator matrix [22]. In [23], an improved PM algorithm is proposed for 2D-DOA estimation, which not only reduces the computational complexity, but also avoids the aperture loss. 

Unfortunately, all the algorithms mentioned above did not consider the characteristics of the impinging signals. In fact, many noncircular signals such as the amplitude modulated (AM), binary phase shift keying (BPSK), minimum shift keying (MSK), and Gaussian MSK (GMSK) signals are used in wireless communication or satellite systems. In recent years, some scholars use non-circular signal characteristics to improve the performance of direction estimation, which contain the noncircular MUSIC (NC-MUSIC) algorithm [24], the NC-ESPRIT algorithm [25], and the noncircular parallel factor (NC-PARAFAC) algorithm [26]. On the one hand, the angle estimation performance can be achieved by the algorithms [24,25,26]. On the other hand, the computation loads are increased greatly due to the doubled array aperture. The noncircular rational invariance propagator method has also been proposed for angle estimation in [27], which aimed at the linear array. If it is extended to the rectangular planar array for 2D-DOA estimation, the complexity would be increased greatly.

In this paper, we take advantage of the characteristics of noncircular signals and derive a novel noncircular propagator method algorithm based on the uniform rectangular planar array. The main works of this paper are listed in a straightforward manner as follows: (1) the property of the noncircular signal and Euler’s transformation are used to construct a new real-valued rectangular array data; (2) the rotational invariance relations for real-valued signal space are depicted in a new way; (3) the PM algorithm is applied to two-dimensional angle estimation for the rectangular planar array which is paired automatically; and (4) theory analysis and simulation results confirm that our algorithm has better direction finding performance and can discern more sources than 2D-PM [23]. Due to real-valued processing, it can save about 75% computational load compared with the NC-PM algorithm [27]. However, its estimation performance is close to NC-PM algorithm, which has higher computational load.

## 2. Data Model 

**Figure 1:**
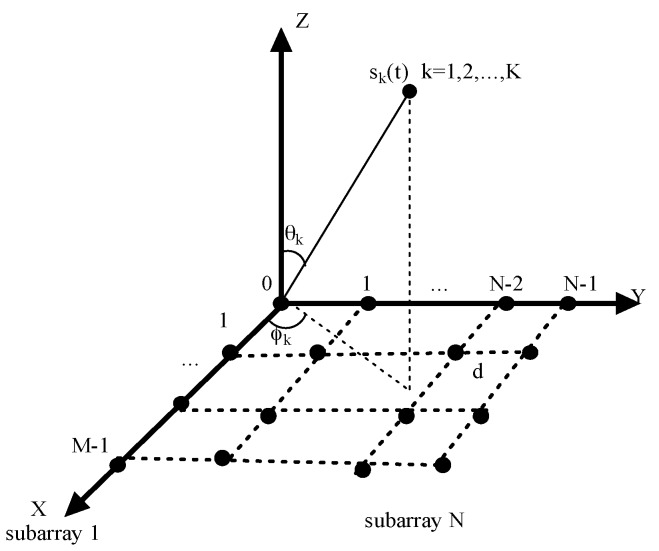
The structure of planar array.

In order to get the two-dimensional direction finding, we consider a uniform rectangular planar array (URA) consisting of N uniform linear subarrays as shown in Figure 1, and there are M sensors in each subarray. The inter-element spacing between the two sensors is d in both the *x*-axis and *y*-axis. Suppose there are K narrowband far-field uncorrelated sources with wavelength λ impinging on the array from different directions. We also assume the noise is independent of the sources and d = λ/2. The output signal of the *i*th subarray $x_{i}(t)$ can be denoted as [26]:

(1) $$x_{i}(t)=A_{x}\Phi^{i-1}S(t)+n_{i}(t),i=1,2,\cdots ,N,$$

where $A_{x}=[a_{x}(v_{1}),a_{x}(v_{2}),\cdots ,a_{x}(v_{K})]$ and $a_{x}(v_{k})={\left[1,e^{-j\pi v_{k}},\cdots ,e^{-j(M-1)\pi v_{k}}\right]}^{T}$, $v_{k}=\cos\phi_{k}\sin\theta_{k}$, $\theta_{k}$ is the elevation angle and $\phi_{k}$ is the azimuth angle. $\Phi =diag(e^{-j\pi u_{1}},e^{-j\pi u_{2}},\cdots ,e^{-j\pi u_{K}})$ and $u_{k}=\sin\phi_{k}\sin\theta_{k}$. $S(t)={\left[s_{1}(t),s_{2}(t),\cdots ,s_{K}(t)\right]}^{T}$ is the noncircular signal vector. In addition, the vector of strictly second-order noncircular signals can be expressed as [28]: $S(t)=\Lambda S_{o}(t)$, $S_{o}(t)\in C^{K\times 1}$, $S_{o}(t)={S_{o}}^{*}(t)$, and $\Lambda =diag\{e^{j\varphi_{1}},e^{j\varphi_{2}},\cdots e^{j\varphi_{K}}\},(e^{j\varphi_{p}}\neq e^{j\varphi_{q}},for\;p\neq q)$. $n_{i}(t)$ is the additive white Gaussian noise vector of the *i*th subarray.

Therefore, the whole array output is

(2) $$x(t)=\left[\begin{array}{c} x_{1}(t)\\ x_{2}(t)\\ \vdots \\ x_{N}(t) \end{array}\right]=\left[\begin{array}{c} A_{x}\\ A_{x}\Phi \\ \vdots \\ A_{x}\Phi^{N-1} \end{array}\right]S(t)+\left[\begin{array}{c} n_{1}(t)\\ n_{2}(t)\\ \vdots \\ n_{N}(t) \end{array}\right]=AS(t)+n(t),$$

where $A=A_{y}\circ A_{x}$ is the MN×K steering vector matrix, ∘ represents the Khatri–Rao product, and $A_{y}=[a_{y}(u_{1}),a_{y}(u_{2}),\cdots ,a_{y}(u_{K})]$, $a_{y}(u_{k})={\left[1,e^{-j\pi u_{k}},\cdots ,e^{-j(N-1)\pi u_{k}}\right]}^{T}$, and $n(t)={\left[n_{1}{\left(t\right)}^{T},n_{2}{\left(t\right)}^{T},\cdots ,n_{N}{\left(t\right)}^{T}\right]}^{T}$.

## 3. Real-Valued PM Algorithm for 2D-DOA Estimation

### 3.1. Euler Transformation

The real part and imaginary part of x(t) can be obtained by utilizing the real-valued property of noncircular signals and Euler’s formula as follows:

(3) $$x_{R}(t)=[x(t)+x^{*}(t)]/2=A_{R}S_{o}(t)+n_{R}(t),$$

(4) $$x_{I}(t)=[x(t)-x^{*}(t)]/(-2j)=A_{I}S_{o}(t)+n_{I}(t),$$

where $n_{R}(t)=[n(t)+n^{*}(t)]/2$, $n_{I}(t)=[n(t)-n^{*}(t)]/(-2j)$,

$$A_{R}=\left[\begin{array}{c} \begin{array}{cccc} \cos\varphi_{1} & \cos\varphi_{2} & \cdots & \cos\varphi_{K}\\ \cos(\pi v_{1}+\varphi_{1}) & \cos(\pi v_{2}+\varphi_{2}) & \cdots & \cos(\pi v_{K}+\varphi_{K})\\ \vdots & \vdots & \vdots & \vdots \\ \cos[\pi (M-1)v_{1}+\varphi_{1}] & \cos[\pi (M-1)v_{2}+\varphi_{2}] & \cdots & \cos[\pi (M-1)v_{K}+\varphi_{K}] \end{array}\}M\\ \begin{array}{cccc} \cos(\pi u_{1}+\varphi_{1}) & \cos(\pi u_{2}+\varphi_{2}) & \cdots & \cos(\pi u_{K}+\varphi_{K})\\ \cos[\pi (v_{1}+u_{1})+\varphi_{1})] & \cos[\pi (v_{2}+u_{2})+\varphi_{2})] & \cdots & \cos[\pi (v_{K}+u_{K})+\varphi_{K})]\\ \vdots & \vdots & \vdots & \vdots \\ \cos[\pi ((M-1)v_{1}+u_{1})+\varphi_{1})] & \cos[\pi ((M-1)v_{2}+u_{2})+\varphi_{2})] & \cdots & \cos[\pi ((M-1)v_{K}+u_{K})+\varphi_{K})] \end{array}\}M\\ \begin{array}{ccccccc} \vdots & \vdots & \vdots & \vdots & \vdots & \vdots & \vdots \end{array}\\ \begin{array}{cccc} \cos[\pi (N-1)u_{1}+\varphi_{1}] & \cos[\pi (N-1)u_{2}+\varphi_{2}] & \cdots & \cos[\pi (N-1)u_{K}+\varphi_{K}]\\ \cos[\pi (N-1)u_{1}+\pi v_{1}+\varphi_{1}] & \cos[\pi (N-1)u_{2}+\pi v_{2}+\varphi_{2}] & \cdots & \cos[\pi (N-1)u_{K}+\pi v_{K}+\varphi_{K}]\\ \vdots & \vdots & \vdots & \vdots \\ \cos[\pi (N-1)u_{1}+\pi (M-1)v_{1}+\varphi_{1}] & \cos[\pi (N-1)u_{2}+\pi (M-1)v_{2}+\varphi_{2}] & & \cos[\pi (N-1)u_{K}+\pi (M-1)v_{K}+\varphi_{K}] \end{array}\}M \end{array}\right]\in R^{MN\times K}$$

$$A_{I}=\left[\begin{array}{c} \begin{array}{cccc} \sin\varphi_{1} & \sin\varphi_{2} & \cdots & \sin\varphi_{K}\\ \sin(\pi v_{1}+\varphi_{1}) & \sin(\pi v_{2}+\varphi_{2}) & \cdots & \sin(\pi v_{K}+\varphi_{K})\\ \vdots & \vdots & \vdots & \vdots \\ \sin[\pi (M-1)v_{1}+\varphi_{1}] & \sin[\pi (M-1)v_{2}+\varphi_{2}] & \cdots & \sin[\pi (M-1)v_{K}+\varphi_{K}] \end{array}\}M\\ \begin{array}{cccc} \sin(\pi u_{1}+\varphi_{1}) & \sin(\pi u_{2}+\varphi_{2}) & \cdots & \sin(\pi u_{K}+\varphi_{K})\\ \sin[\pi (v_{1}+u_{1})+\varphi_{1}] & \sin[\pi (v_{2}+u_{2})+\varphi_{2}] & \cdots & \sin[\pi (v_{K}+u_{K})+\varphi_{K}]\\ \vdots & \vdots & \vdots & \vdots \\ \sin[\pi ((M-1)v_{1}+u_{1})+\varphi_{1}] & \sin[\pi ((M-1)v_{2}+u_{2})+\varphi_{2}] & \cdots & \sin[\pi ((M-1)v_{K}+u_{K})+\varphi_{K}] \end{array}\}M\\ \begin{array}{ccccccc} \vdots & \vdots & \vdots & \vdots & \vdots & \vdots & \vdots \end{array}\\ \begin{array}{cccc} \sin[\pi (N-1)u_{1}+\varphi_{1}] & \sin[\pi (N-1)u_{2}+\varphi_{2}] & \cdots & \sin[\pi (N-1)u_{K}+\varphi_{K}]\\ \sin[\pi (N-1)u_{1}+\pi v_{1}+\varphi_{1}] & \sin[\pi (N-1)u_{2}+\pi v_{2}+\varphi_{2}] & \cdots & \sin[\pi (N-1)u_{K}+\pi v_{K}+\varphi_{K}]\\ \vdots & \vdots & \vdots & \vdots \\ \sin[\pi (N-1)u_{1}+\pi (M-1)v_{1}+\varphi_{1}] & \sin[\pi (N-1)u_{2}+\pi (M-1)v_{2}+\varphi_{2}] & & \sin[\pi (N-1)u_{K}+\pi (M-1)v_{K}+\varphi_{K}] \end{array}\}M \end{array}\right]\in R^{MN\times K}$$

Then, we define a new virtual array data as follows:

(5) $$y(t)=\left[\begin{array}{c} x_{R}(t)\\ x_{I}(t) \end{array}\right]=BS_{o}(t)+n_{r}(t),$$

where $B=\left[\begin{array}{c} A_{R}\\ A_{I} \end{array}\right]\in R^{2MN\times K}$, $n_{r}(t)=\left[\begin{array}{c} n_{R}(t)\\ n_{I}(t) \end{array}\right]\in R^{2MN\times 1}$. 

Define two matrices as follows: $T_{1}=[I_{M(N-1)}0_{M(N-1)\times M}]\in R^{M(N-1)\times MN}$, $T_{2}=[0_{M(N-1)\times M}I_{M(N-1)}]$ $\in R^{M(N-1)\times MN}$; then, we construct two matrices $J_{1}=[T_{1}+T_{2},\;0_{M(N-1)\times MN}]\in R^{M(N-1)\times 2MN}$ and $J_{2}=[0_{M(N-1)\times MN},T_{2}-T_{1}]\in R^{M(N-1)\times 2MN}$, and we can get the following relationship:

(6) $$J_{2}B=J_{1}BG,$$

where $G=diag\left\{\tan(\frac{\pi u_{1}}{2}),\tan(\frac{\pi u_{2}}{2}),\cdots ,\tan(\frac{\pi u_{K}}{2})\right\}$ is a real-valued matrix whose diagonal elements contain the needed angle information:

$$J_{1}B=\left[\begin{array}{c} \begin{array}{cccc} 2\cos\frac{\pi u_{1}+2\phi_{1}}{2}\cos\frac{\pi u_{1}}{2} & 2\cos\frac{\pi u_{2}+2\phi_{2}}{2}\cos\frac{\pi u_{2}}{2} & \cdots & 2\cos\frac{\pi u_{K}+2\phi_{K}}{2}\cos\frac{\pi u_{K}}{2}\\ 2\cos\frac{2\pi v_{1}+\pi u_{1}+2\phi_{1}}{2}\cos\frac{\pi u_{1}}{2} & 2\cos\frac{2\pi v_{2}+\pi u_{2}+2\phi_{2}}{2}\cos\frac{\pi u_{2}}{2} & \cdots & 2\cos\frac{2\pi v_{K}+\pi u_{K}+2\phi_{K}}{2}\cos\frac{\pi u_{K}}{2}\\ \vdots & \vdots & \vdots & \vdots \\ 2\cos\frac{2\pi (M-1)v_{1}+\pi u_{1}+2\phi_{1}}{2}\cos\frac{\pi u_{1}}{2} & 2\cos\frac{2\pi (M-1)v_{2}+\pi u_{2}+2\phi_{2}}{2}\cos\frac{\pi u_{2}}{2} & \cdots & 2\cos\frac{2\pi (M-1)v_{K}+\pi u_{K}+2\phi_{K}}{2}\cos\frac{\pi u_{K}}{2} \end{array}\}M\\ \begin{array}{cccc} 2\cos\frac{3\pi u_{1}+2\phi_{1}}{2}\cos\frac{\pi u_{1}}{2} & 2\cos\frac{3\pi u_{2}+2\phi_{2}}{2}\cos\frac{\pi u_{2}}{2} & \cdots & 2\cos\frac{3\pi u_{K}+2\phi_{K}}{2}\cos\frac{\pi u_{K}}{2}\\ 2cos\frac{3\pi u_{1}+2\pi v_{1}+2\phi_{1}}{2}\cos\frac{\pi u_{1}}{2} & 2cos\frac{3\pi u_{2}+2\pi v_{2}+2\phi_{2}}{2}\cos\frac{\pi u_{2}}{2} & \cdots & 2cos\frac{3\pi u_{K}+2\pi v_{K}+2\phi_{K}}{2}\cos\frac{\pi u_{K}}{2}\\ \vdots & \vdots & \vdots & \vdots \\ 2cos\frac{3\pi u_{1}+2\pi (M-1)v_{1}+2\phi_{1}}{2}\cos\frac{\pi u_{1}}{2} & 2cos\frac{3\pi u_{2}+2\pi (M-1)v_{2}+2\phi_{2}}{2}\cos\frac{\pi u_{2}}{2} & \cdots & 2cos\frac{3\pi u_{K}+2\pi (M-1)v_{K}+2\phi_{K}}{2}\cos\frac{\pi u_{K}}{2} \end{array}\}M\\ \begin{array}{ccccccc} \vdots & \vdots & \vdots & \vdots & \vdots & \vdots & \vdots \end{array}\\ \begin{array}{cccc} 2\cos\frac{\pi (2N-3)u_{1}+2\phi_{1}}{2}\cos\frac{\pi u_{1}}{2} & 2\cos\frac{\pi (2N-3)u_{2}+2\phi_{2}}{2}\cos\frac{\pi u_{2}}{2} & \cdots & 2\cos\frac{\pi (2N-3)u_{K}+2\phi_{K}}{2}\cos\frac{\pi u_{K}}{2}\\ 2\cos\frac{\pi (2N-3)u_{1}+2\pi v_{1}+2\phi_{1}}{2}\cos\frac{\pi u_{1}}{2} & 2\cos\frac{\pi (2N-3)u_{2}+2\pi v_{2}+2\phi_{2}}{2}\cos\frac{\pi u_{2}}{2} & \cdots & 2\cos\frac{\pi (2N-3)u_{K}+2\pi v_{K}+2\phi_{K}}{2}\cos\frac{\pi u_{K}}{2}\\ \vdots & \vdots & \vdots & \vdots \\ 2\cos\frac{\pi (2N-3)u_{1}+2\pi (M-1)v_{1}+2\phi_{1}}{2}\cos\frac{\pi u_{1}}{2} & 2\cos\frac{\pi (2N-3)u_{2}+2\pi (M-1)v_{2}+2\phi_{2}}{2}\cos\frac{\pi u_{2}}{2} & & 2\cos\frac{\pi (2N-3)u_{K}+2\pi (M-1)v_{K}+2\phi_{K}}{2}\cos\frac{\pi u_{K}}{2} \end{array}\}M \end{array}\right]\in R^{M(N-1)\times K}$$

$$J_{2}B=\left[\begin{array}{c} \begin{array}{cccc} 2\cos\frac{\pi u_{1}+2\phi_{1}}{2}sin\frac{\pi u_{1}}{2} & 2\cos\frac{\pi u_{2}+2\phi_{2}}{2}\cos\frac{\pi u_{2}}{2} & \cdots & 2\cos\frac{\pi u_{K}+2\phi_{K}}{2}sin\frac{\pi u_{K}}{2}\\ 2\cos\frac{2\pi v_{1}+\pi u_{1}+2\phi_{1}}{2}sin\frac{\pi u_{1}}{2} & 2\cos\frac{2\pi v_{2}+\pi u_{2}+2\phi_{2}}{2}sin\frac{\pi u_{2}}{2} & \cdots & 2\cos\frac{2\pi v_{K}+\pi u_{K}+2\phi_{K}}{2}sin\frac{\pi u_{K}}{2}\\ \vdots & \vdots & \vdots & \vdots \\ 2\cos\frac{2\pi (M-1)v_{1}+\pi u_{1}+2\phi_{1}}{2}sin\frac{\pi u_{1}}{2} & 2\cos\frac{2\pi (M-1)v_{2}+\pi u_{2}+2\phi_{2}}{2}sin\frac{\pi u_{2}}{2} & \cdots & 2\cos\frac{2\pi (M-1)v_{K}+\pi u_{K}+2\phi_{K}}{2}sin\frac{\pi u_{K}}{2} \end{array}\}M\\ \begin{array}{cccc} 2\cos\frac{3\pi u_{1}+2\phi_{1}}{2}sin\frac{\pi u_{1}}{2} & 2\cos\frac{3\pi u_{2}+2\phi_{2}}{2}sin\frac{\pi u_{2}}{2} & \cdots & 2\cos\frac{3\pi u_{K}+2\phi_{K}}{2}\sin\frac{\pi u_{K}}{2}\\ 2cos\frac{3\pi u_{1}+2\pi v_{1}+2\phi_{1}}{2}sin\frac{\pi u_{1}}{2} & 2cos\frac{3\pi u_{2}+2\pi v_{2}+2\phi_{2}}{2}sin\frac{\pi u_{2}}{2} & \cdots & 2cos\frac{3\pi u_{K}+2\pi v_{K}+2\phi_{K}}{2}sin\frac{\pi u_{K}}{2}\\ \vdots & \vdots & \vdots & \vdots \\ 2cos\frac{3\pi u_{1}+2\pi (M-1)v_{1}+2\phi_{1}}{2}sin\frac{\pi u_{1}}{2} & 2cos\frac{3\pi u_{2}+2\pi (M-1)v_{2}+2\phi_{2}}{2}sin\frac{\pi u_{2}}{2} & \cdots & 2cos\frac{3\pi u_{K}+2\pi (M-1)v_{K}+2\phi_{K}}{2}sin\frac{\pi u_{K}}{2} \end{array}\}M\\ \begin{array}{ccccccc} \vdots & \vdots & \vdots & \vdots & \vdots & \vdots & \vdots \end{array}\\ \begin{array}{cccc} 2\cos\frac{\pi (2N-3)u_{1}+2\phi_{1}}{2}sin\frac{\pi u_{1}}{2} & 2\cos\frac{\pi (2N-3)u_{2}+2\phi_{2}}{2}sin\frac{\pi u_{2}}{2} & \cdots & 2\cos\frac{\pi (2N-3)u_{K}+2\phi_{K}}{2}sin\frac{\pi u_{K}}{2}\\ 2\cos\frac{\pi (2N-3)u_{1}+2\pi v_{1}+2\phi_{1}}{2}sin\frac{\pi u_{1}}{2} & 2\cos\frac{\pi (2N-3)u_{2}+2\pi v_{2}+2\phi_{2}}{2}sin\frac{\pi u_{2}}{2} & \cdots & 2\cos\frac{\pi (2N-3)u_{K}+2\pi v_{K}+2\phi_{K}}{2}sin\frac{\pi u_{K}}{2}\\ \vdots & \vdots & \vdots & \vdots \\ 2\cos\frac{\pi (2N-3)u_{1}+2\pi (M-1)v_{1}+2\phi_{1}}{2}sin\frac{\pi u_{1}}{2} & 2\cos\frac{\pi (2N-3)u_{2}+2\pi (M-1)v_{2}+2\phi_{2}}{2}sin\frac{\pi u_{2}}{2} & & 2\cos\frac{\pi (2N-3)u_{K}+2\pi (M-1)v_{K}+2\phi_{K}}{2}sin\frac{\pi u_{K}}{2} \end{array}\}M \end{array}\right]\in R^{M(N-1)\times K}.$$

Similarly, define two (M−1)×M Toeplitz matrices as follows: $T_{3}=\left[\begin{array}{cccccc} 1 & 1 & 0 & 0 & \cdots & 0\\ 0 & 1 & 1 & 0 & \cdots & 0\\ \vdots & \vdots & \vdots & \vdots & \ddots & \vdots \\ 0 & \cdots & 0 & 0 & 1 & 1 \end{array}\right]$, $T_{4}=\left[\begin{array}{cccccc} 1 & -1 & 0 & 0 & \cdots & 0\\ 0 & 1 & -1 & 0 & \cdots & 0\\ \vdots & \vdots & \vdots & \vdots & \ddots & \vdots \\ 0 & \cdots & 0 & 0 & 1 & -1 \end{array}\right]$.

Then, we construct two matrices $J_{3}$ and $J_{4}$ as follows: $J_{3}=I_{2N}⊗T_{3}\in R^{2(M-1)N\times 2MN}$ and $J_{4}=\left[\begin{array}{cc} 0 & -I_{N}⊗T_{4}\\ I_{N}⊗T_{4} & 0 \end{array}\right]\in R^{2(M-1)N\times 2MN}$.

We also get the following relationship:

(7) $$J_{4}B=J_{3}BD,$$

where $D=diag\left\{\tan(\frac{\pi v_{1}}{2}),\tan(\frac{\pi v_{2}}{2}),\cdots ,\tan(\frac{\pi v_{K}}{2})\right\}$ is a real-valued matrix whose diagonal elements also contain the desired angle information.

### 3.2. 2D-DOA Estimation

According to Equation (5), the estimation of covariance matrix R of y(t) is denoted by collecting *L* snapshots:

(8) $$R=\frac{1}{L}\sum_{i=1}^{L}y(t_{i})y^{H}(t_{i}).$$

From Equation (8), R can be denoted by $R=\left[\begin{array}{c} R_{x1}\\ R_{x2} \end{array}\right]$, where $R_{x1}\in R^{K\times 2MN}$, $R_{x2}\in R^{(2MN-K)\times 2MN}$. In the noiseless case, $R_{x1}p_{r}=R_{x2}$, an estimation matrix $p_{r}$ can be obtained by [21]:

(9) $${\hat{p}}_{r}={\left(R_{x1}R_{x1}^{H}\right)}^{-1}R_{x1}R_{x2}^{H}.$$

We construct a new matrix $p_{s}=\left[\begin{array}{c} I_{K}\\ {{\hat{P}}_{r}}^{H} \end{array}\right]$, where $I_{K}$ is the identity matrix. In the noiseless case, the relationship between $p_{s}$ and B can be obtained by a unique non-singular matrix T as

(10) $$p_{s}=BT.$$

Substituting Equation (10) into Equation (6), we can get

(11) $$J_{2}p_{s}T^{-1}=J_{1}p_{s}T^{-1}G.$$

If we define $P_{1}={\left(J_{1}p_{s}\right)}^{†}J_{2}p_{s}$, we then have

(12) $$P_{1}=T^{-1}GT.$$

Equation (12) shows that the diagonal elements of the matrix G can be obtained by performing the eigenvalue decomposition of $P_{1}$, and T is the corresponding eigenvector.

Then, we can get the estimation of ${\hat{u}}_{k}$:

(13) $${\hat{u}}_{k}=2\arctan({\hat{p}}_{k})/\pi ,$$

where ${\hat{p}}_{k}$ is the *k*th diagonal element of the matrix G.

Similarly, Substituting Equation (10) into Equation (7), we can also get

(14) $$J_{4}p_{s}T^{-1}=J_{3}p_{s}T^{-1}D.$$

If we define $P_{2}={\left(J_{4}p_{s}\right)}^{†}J_{3}p_{s}$, we then have

(15) $$P_{2}=T^{-1}DT.$$

Then, we get the estimation of ${\hat{v}}_{k}$:

(16) $${\hat{v}}_{k}=2\arctan({\hat{r}}_{k})/\pi ,$$

where ${\hat{r}}_{k}$ is the *k*th diagonal element of the matrix D.

We note that ${\hat{u}}_{k}$ and ${\hat{v}}_{k}$ share the same eigenvector T, so the pairing is automatically formed. Thus, 2D-DOA can be obtained by

(17) $${\hat{\theta }}_{k}=\sin^{-1}\left(\sqrt{{{\hat{u}}_{k}}^{2}+{{\hat{v}}_{k}}^{2}}\right),$$

(18) $${\hat{\phi }}_{k}=\tan^{-1}\left({\hat{u}}_{k}/{\hat{v}}_{k}\right).$$

We have now achieved the essence of the proposed algorithm. The major algorithmic steps are as follows:

(1) Construct the matrix y(t) from Equation (5), and compute the covariance matrix R of y(t) through Equation (8).
(2) Estimation of the propagator $p_{r}$ from Equation (9), and then construct the matrix $p_{s}$.
(3) Construct the matrix $J_{1}p_{s}$ and $J_{2}p_{s}$ and perform the eigenvalue decomposition of $P_{1}={\left(J_{1}p_{s}\right)}^{†}J_{2}p_{s}$.
(4) Similarly, construct the matrix $J_{3}p_{s}$ and $J_{4}p_{s}$ and perform the eigenvalue decomposition of $P_{2}={\left(J_{3}p_{s}\right)}^{†}{\tilde{J}}_{4}p_{s}$.
(5) Finally, estimate the 2D-DOA through Equations (17) and (18).

**Remark** **1.** In [23], the conventional PM algorithm divides the steering matrix A into two matrices $A_{1}\in C^{K\times K}$ and $A_{2}\in C^{(MN-K)\times K}$, and $A_{2}$ is the linear transformation of $A_{1}$, i.e., $A_{2}={P_{m}}^{H}A_{1}$, $P_{m}\in C^{K\times (MN-K)}$ is the propagator operator. According to Equation (1), x(t)=AS(t)+n(t), and the covariance matrix of received data $x(t)\in C^{MN\times 1}$ is $R_{p}=\frac{1}{L}\sum_{i=1}^{L}x(t_{i})x^{H}(t_{i})$. We partition it as $R_{p}=\left[\begin{array}{c} R_{p1}\\ R_{p2} \end{array}\right]$, where $R_{p1}\in R^{K\times MN}$, $R_{p2}\in R^{(MN-K)\times MN}$, and we can get the propagator estimator ${\hat{p}}_{m}={\left(R_{p1}R_{p1}^{H}\right)}^{-1}R_{p1}R_{p2}^{H}$. In our paper, according to Equation (5), $y(t)=\left[\begin{array}{c} x_{R}(t)\\ x_{I}(t) \end{array}\right]=BS_{o}(t)+n_{r}(t)$, and we compute the covariance of $y(t)\in R^{2MN\times 1}$ to estimate the propagator. Apparently, the available array aperture of the proposed algorithm can be thought of as twice that of the conventional 2D-PM [23], so it has better angle performance than 2D-PM.

**Remark** **2.** In [23], define $p_{c}=\left[\begin{array}{c} I_{K}\\ {{\hat{P}}_{m}}^{H} \end{array}\right]$, and then $p_{c}A_{1}=A$, which means that the columns in $p_{c}$ span the same signal subspace as the column vectors in A. Divide $p_{c}$ into $p_{c_{1}}\in C^{M(N-1)\times K}$ and $p_{c_{2}}\in C^{M(N-1)\times K}$, $p_{c_{1}}$, $p_{c_{2}}$ are the first M(N−1) rows and the last M(N−1) rows of $p_{c}$. Then, get the relationship, $P_{c1}^{+}P_{c2}=A_{1}\Phi A_{1}$, where $\Phi =diag(e^{-j\pi u_{1}},e^{-j\pi u_{2}},\cdots ,e^{-j\pi u_{K}})$. Perform the eigenvalue decomposition of $P_{c1}^{+}P_{c2}$ to obtain the diagonal elements of the matrix Φ. Similarly, reconstruct $p_{c}$ to $p_{c}^{\prime }$, $p_{c_{1}}^{\prime }$, $p_{c_{2}}^{\prime }$ being the first N(M−1) rows and the last N(M−1) rows of $p_{c}^{\prime }$, and perform the eigenvalue decomposition of $P_{c1}^{\prime }{}^{+}P_{c2}^{\prime }$ to obtain the diagonal elements of the matrix $\Phi_{x}$, where $\Phi_{x}=diag(e^{-j\pi v_{1}},e^{-j\pi v_{2}},\cdots ,e^{-j\pi v_{K}})$. Finally, the 2D-DOA can be obtained from the diagonal elements of Φ and $\Phi_{x}$. From the above mentioned, the row dimensions of $p_{c_{1}}$, $p_{c_{2}}$ and $p_{c_{1}}^{\prime }$, $p_{c_{2}}^{\prime }$ are equal to M(N−1),(M−1)N, respectively. The maximum number of the identified sources is min[M(N−1),(M−1)N]. In our proposed algorithm, from Equation (11) and Equation (14), the row dimensions of $J_{1}p_{s}$ and $J_{2}p_{s}$, $J_{3}p_{s}$ and $J_{4}p_{s}$ are equal to M(N−1),2(M−1)N, respectively. Therefore, the maximum number of the identified sources is min [M(N−1),2(M−1)N]. If M < N, the proposed algorithm can discern more sources than that of the conventional 2D-PM [23].

**Remark** **3.** *In the NC-PM algorithm [27], according to Equation (2), the extended array output data is denoted as* $Y=\left[\begin{array}{c} X\\ J_{MN}X^{*} \end{array}\right]=A_{nc}S_{o}+N_{nc}$*, where* $A_{nc}\in C^{2MN\times K}$*,* $J_{MN}$ *is the* MN×MN *exchange matrix with ones on its anti-diagnoal and zeros elsewhere, and* $X^{*}\in C^{MN\times L}$ *stands for the complex conjugation of* X*,* $Y\in C^{2MN\times L}$*. Compute the covariance of* Y *to estimate the propagator* $p_{nc}$*. Similarly, the invariance equations for* $p_{nc}$ *are constructed to estimate the 2D-DOA. As is known to us, each computation amount of the complex multiplication is four times that of the real-valued one. In our algorithm, we use Euler transformation to convert complex arithmetic of noncircular to real arithmetic. For example, according to Equation (5),* $y(t)=BS_{o}(t)+n_{r}(t)$*,* $y(t)\in R^{2MN\times 1}$*, and the computation amounts of covariance of* y(t) *with snapshots L are much lower than that of Y [27]. Due to real-valued processing, our algorithm can save about 75% computational load compared with the NC-PM algorithm [27].*

## 4. Cramer-Rao Bounds and Analysis

### 4.1. CRB

In this section, we give the Cramer-Rao Bounds (CRB) of noncircular signal for rectangular planar array. According to Equation (5), the received data is

(19) $$y(t)=\left[\begin{array}{c} x_{R}(t)\\ x_{I}(t) \end{array}\right]=BS_{o}(t)+n_{r}(t),$$

where $B=[b_{1},b_{2},\cdots ,b_{K}]\in C^{2MN\times K}$, and $n_{r}(t)$ is the noise vector. The Fisher information matrix (FIM) in relation to $\phi =[\phi_{1},\phi_{2},\cdots ,\phi_{K}]$ and $\theta =[\theta_{1},\theta_{2},\cdots ,\theta_{K}]$ can be calculated as follows [29]:

(20) $$F=\left[\begin{array}{cc} F_{11} & F_{12}\\ F_{21} & F_{22} \end{array}\right].$$

According to [29], we know that the (i,j) *i*th element of $F_{11}$ is given by

(21) F(θi,θj)=2Re[trace(B˙θiSo)HΓ−1(B˙θjSo)]=2Re[trace(B˙θeieiTSo)HΓ−1(B˙θejejTSo)]=Re[trace(SoHeieiTB˙θHΓ−1B˙θejejTSo)]=Re[trace(eiTB˙θHΓ−1B˙θej)(ejTSoSoHei)]=2LRe[(B˙θHΓ−1B˙θ)ij(RsoT)ij].

Likely, we can give the (i,j) *i*th element of $F_{12}$, $F_{21}$, $F_{22}$:

(22) F(θi,ϕj)=2LRe[(B˙θHΓ−1B˙ϕ)ij(RsoT)ij],

(23) F(ϕi,θj)=2LRe[(B˙ϕHΓ−1B˙θ)ij(RsoT)ij],

(24) F(ϕi,ϕj)=2LRe[(B˙ϕHΓ−1B˙ϕ)ij(RsoT)ij],

where $e_{i}$ denotes the *i*th column of the unit matrix, $R_{s_{o}}=\frac{1}{L}S_{o}S_{o}^{H}$, ${\dot{B}}_{\varsigma_{i}}=\frac{\partial B}{\partial \varsigma_{i}}$, ${\dot{B}}_{\xi }=[\frac{\partial B}{\partial \xi_{1}},\frac{\partial B}{\partial \xi_{2}},\cdots ,\frac{\partial B}{\partial \xi_{K}}]$, $\Gamma =\left[\begin{array}{cccc} \sigma^{2}{Ι}_{2MN} & 0 & \cdots & 0\\ 0 & \sigma^{2}{Ι}_{2MN} & \cdots & 0\\ \vdots & \vdots & \ddots & \vdots \\ 0 & 0 & \cdots & \sigma^{2}{Ι}_{2MN} \end{array}\right]$, $\sigma^{2}$ is the covariance of the noise. According to Equations (21)–(24), we can obtain:

(25) F11=2LRe[(B˙θHΓ−1B˙θ)⊕(RsoT)],

(26) F12=2LRe[(B˙θHΓ−1B˙ϕ)⊕(RsoT)],

(27) F21=2LRe[(B˙ϕHΓ−1B˙θ)⊕(RsoT)],

(28) F22=2LRe[(B˙ϕHΓ−1B˙ϕ)⊕(RsoT)],

where ⊕ represents Hadamard product.

Then, the CRB can be denoted as:

(29) $$\mathrm{CRB}=F^{-1}.$$

**Figure 2:**
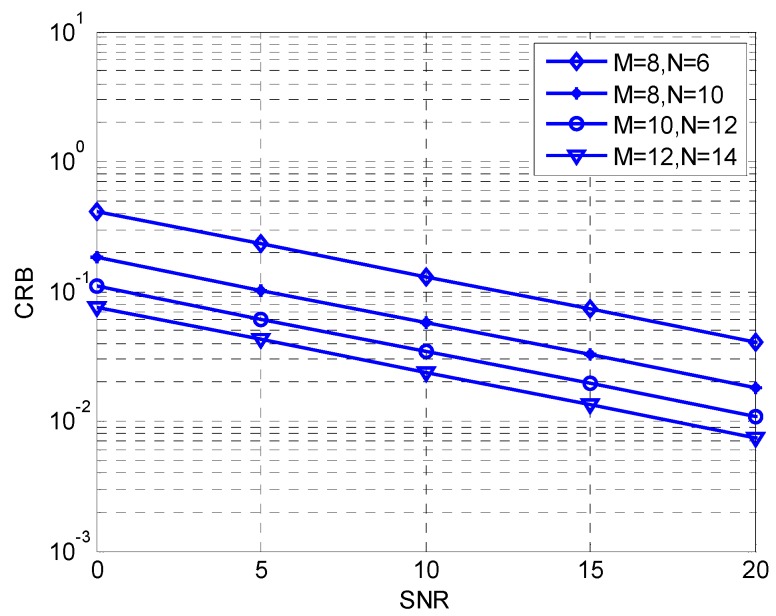
CRB comparison versus SNR.

**Figure 3:**
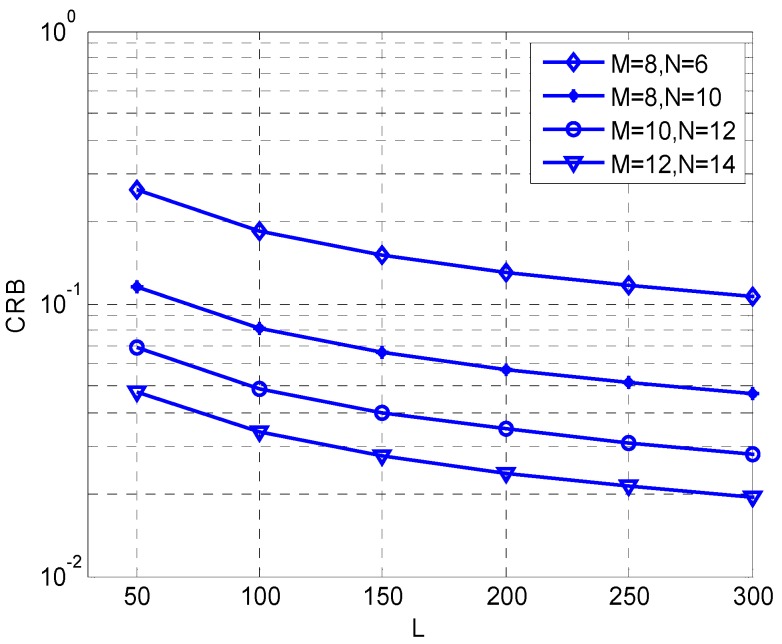
CRB comparison versus snapshots *L*.

We present the curves of CRB versus different signal to noise ratios (SNRs) and snapshots *L* in Figure 2 and Figure 3. The source number *K* is fixed at 3 *M* and *N* represents the numbers of sensors on the *x*-axis and the *y*-axis. In Figure 2, the snapshot *L* is fixed at 200. It is obvious that, with the improvement of SNR, the value of CRB decreases accordingly. In Figure 3, we set SNR at 20 dB, and the curve shows that the value of CRB decreases with increase of *L*, and simulation results and theory analysis are consistent.

### 4.2. Complexity Analysis

In this section, we analyse the computational complexity of the algorithm specifically. First, estimation of the covariance matrix R requires $O(4M^{2}N^{2}L)$ real-valued multiplications (RMS). In addition, the estimation of the matrix $p_{s}$ takes $O(2MNK^{2}+4M^{2}N^{2}K+K^{3})$ RMS. Then, the estimation and eigenvalue decomposition of the matrix $P_{1}$ and $P_{2}$ totally require $O(4M^{2}NK(N-1)+3M(N-1)K^{2}+8M(M-1)N^{2}K+2K^{3})$ RMS. Therefore, the overall computational complexity of our algorithm is $O(4M^{2}N^{2}L+5MNK^{2}+16M^{2}N^{2}K+3K^{3}-4M^{2}NK-3MK^{2}-8MN^{2}K)$ RMS. As we know that each computation amount of the complex multiplication is four times that of the real-valued one, we can show the Chen’s noncircular propagator algorithm [27] needs $O(16M^{2}N^{2}L+8MNK^{2}+16M^{2}N^{2}K+3K^{3}+16M(N-1)K^{2})$ RMS, J’s noncircular ESPRIT [25] needs $O(16M^{2}N^{2}L+M^{3}N^{3}+8M(N-1)K^{2}+3K^{3}+8N(M-1)K^{2})$ RMS, Zhang’s 2D-ESPRIT algorithm [8] needs $O(4M^{2}N^{2}L+M^{3}N^{3}+4M(N-1)K^{2}+4N(M-1)K^{2}+6K^{3})$ RMS, while Li’s 2D-PM [23] requires $O(4M^{2}N^{2}L+4MNK^{2}+4M^{2}N^{2}K+6K^{3}+4M(N-1)K^{2}+4N(M-1)K^{2})$ RMS. 

**Figure 4:**
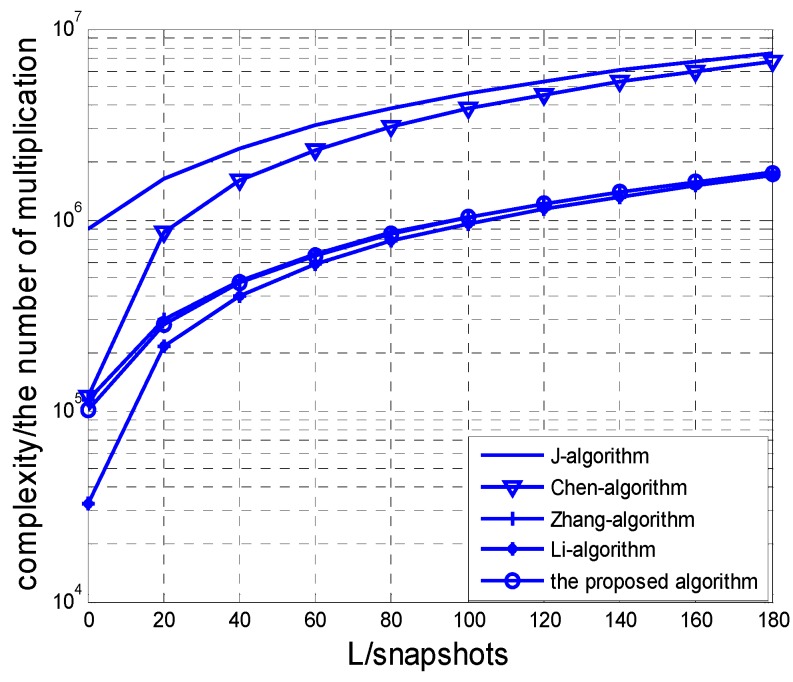
Complexity comparison versus *L.*

**Figure 5:**
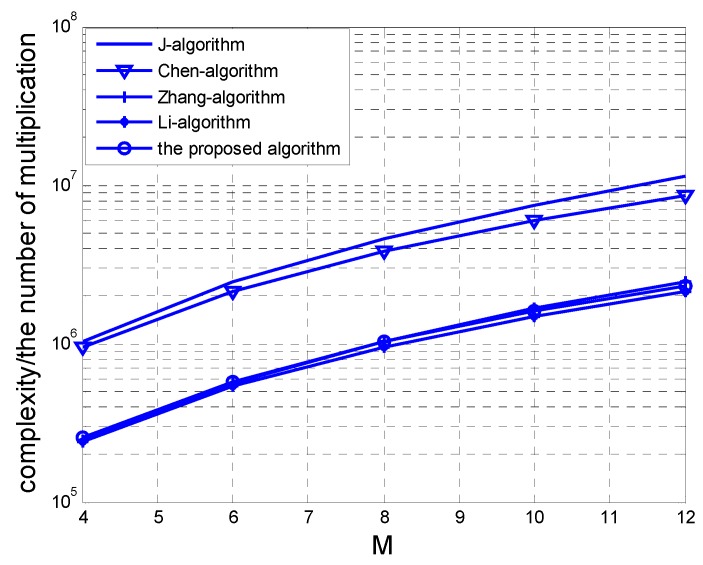
Complexity comparison versus *M*.

The complexity comparisons with different parameters are shown in Figure 4 and Figure 5. In Figure 4, the numbers of sensor *M* and *N* on the *x*-axis and the *y*-axis are set at 8 and 6, respectively. The source number *K* is fixed at 3. In Figure 5, the parameters *N* and *K* are the same as Figure 4, and the snapshot *L* is set to 100. From Figure 4 and Figure 5, we can observe that the proposed algorithm has much lower computational load than J’s NC-ESPRIT algorithm and Chen’s NC-PM algorithm.

We can summarize the merits of the proposed algorithm as follows:

(1) The proposed algorithm has much lower computational load than the NC-PM and NC-ESPRIT algorithms because the proposed algorithm uses Euler transformation to convert complex arithmetic of noncircular PM to real arithmetic.
(2) The proposed algorithm has better estimation performance than the 2D-PM algorithm because the array aperture is doubled according to Equation (5).
(3) The maximum number of discerned sources of our algorithm is dependent on Equation (5) and the real-valued PM method. Obviously, the maximum number of the identified sources of our proposed algorithm is min[M(N−1),2(M−1)N], while 2D-PM is min[M(N−1),(M−1)N].
(4) The proposed algorithm requires no extra matching calculation. The estimated 2D-DOA can automatically be matched.

## 5. Simulation Results

**Figure 6:**
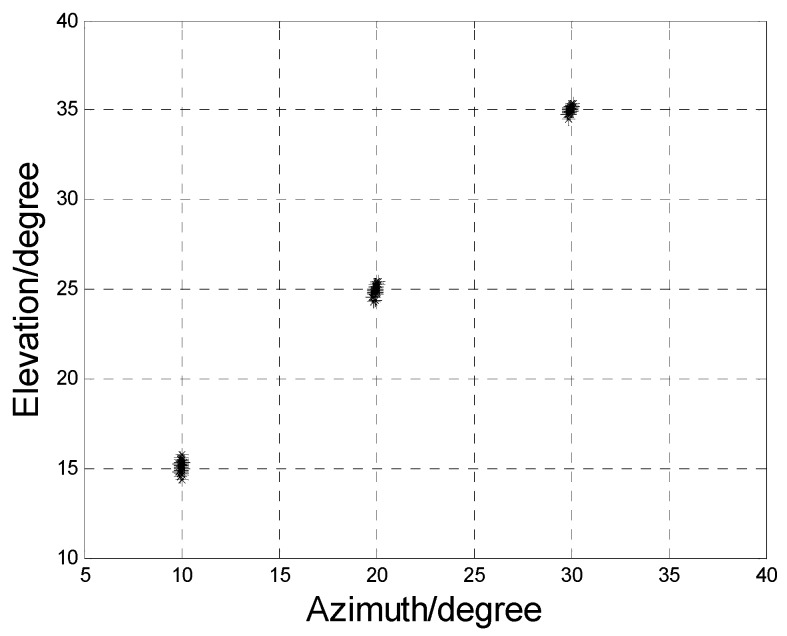
Angle estimation results.

**Figure 7:**
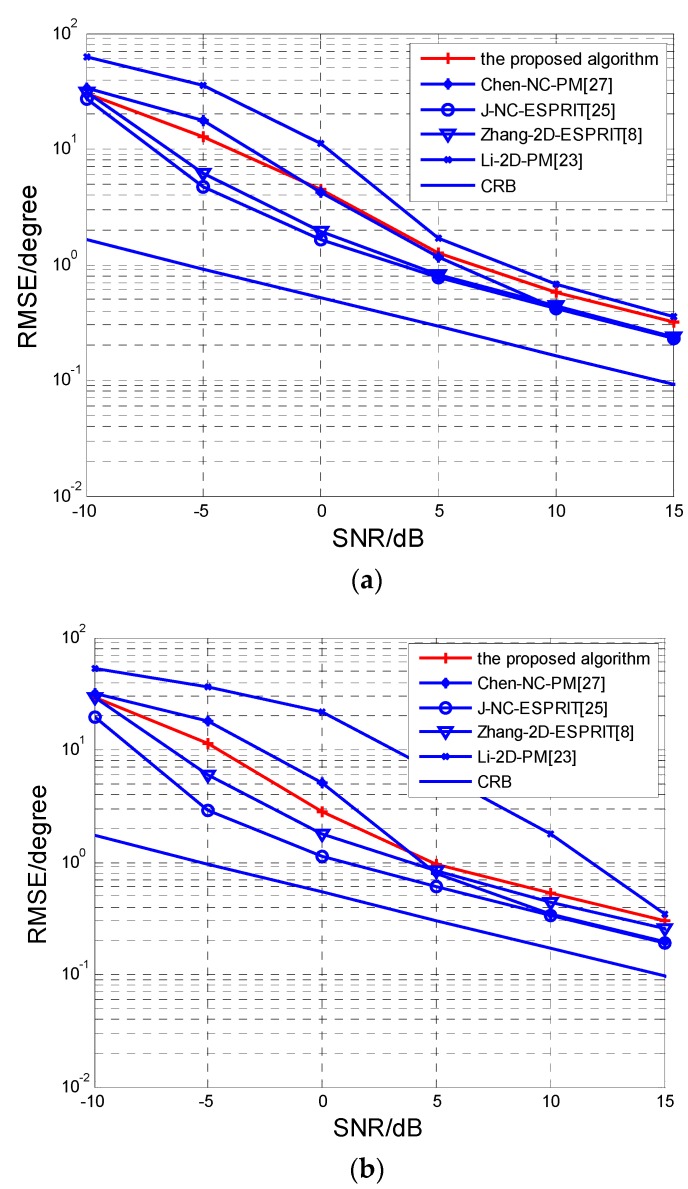
The root mean squared error (RMSE) comparison of different algorithms versus SNR. (**a**) *M* = 6, *N* = 8, *L* = 100; (**b**) *M* = 8, *N* = 8, *L* = 50.

**Figure 8:**
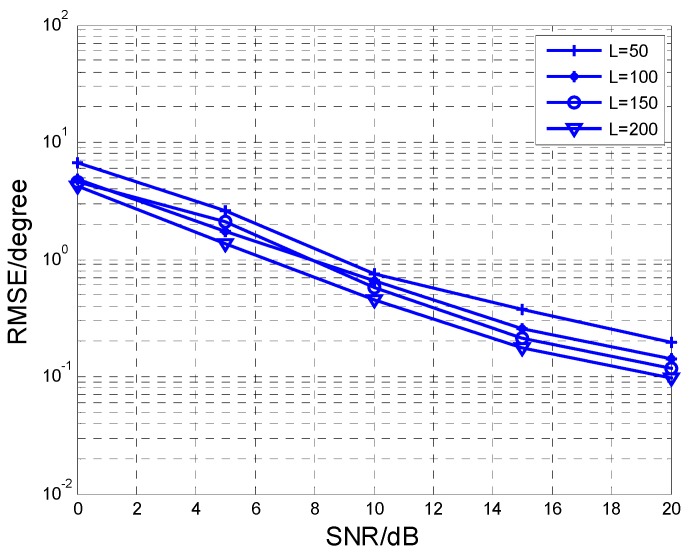
RMSE comparison at different values of *L*.

In this section, we use Monte Carlo simulations to verify the performance of the algorithm. In the simulation, the rectangular planar array is configured with *N* subarrays, each subarray contains *M* sensors, *L* is the snapshots of the sources, and *K* is the number of the sources. We assume that there are *K* = 3 non-coherent sources, which are BPSK modulated in Figure 4, Figure 5, Figure 6, Figure 7 and Figure 8, where $\left(\theta_{1},\phi_{1})=(15^{\circ },10^{\circ }\right)$, $\left(\theta_{2},\phi_{2})=(25^{\circ },20^{\circ }\right)$ and $\left(\theta_{3},\phi_{3})=(35^{\circ },30^{\circ }\right)$, respectively. 

The root mean squared error (RMSE) is used for performance assessment, which is defined as $\frac{1}{K}\sum_{k=1}^{K}\sqrt{\frac{1}{1000}\sum_{n=1}^{1000}{\left({\hat{\theta }}_{k,n}-\theta_{k}\right)}^{2}+{\left({\hat{\phi }}_{k,n}-\phi_{k}\right)}^{2}}$, where ${\hat{\theta }}_{k,n}$, ${\hat{\phi }}_{k,n}$ are the estimated value of $\theta_{k}$ and $\phi_{k}$ for the *n*th trial.

Figure 6 gives the angle pairing results of the proposed algorithm with 50 Monte Carlo trials, where M=8, N=6, L=200 and SNR is 10 dB. From Figure 4, we can observe that the 2D-DOAs of all three sources are localized clearly and paired automatically, which proves the effectiveness of our algorithm.

Figure 7a,b presents RMSE comparison at different SNRs among the proposed algorithm, J’s NC-ESPRIT algorithm [25], Chen’s NC-PM algorithm [27], Zhang’s 2D-ESPRIT algorithm [8], Li’s 2D-PM algorithm [23] and CRB. In Figure 5a, we set *M* = 6, *N* = 8, *L* = 100. In Figure 5b, we change the numbers of sensors and snapshots and set *M* = 8, *N* = 8, and *L* = 50. From the curves of Figure 5a,b, we know that the proposed algorithm has better RMSE performance than Li’s algorithm [23]. Furthermore, it has close RMSE performance to Chen’s algorithm [27]. However, we should know that our algorithm has much lower computational amount than J’s NC-ESPRIT algorithm and Chen’s NC-PM algorithm owing to the real-valued processing, which means that it is more suitable for a practical application system.

Figure 8 presents RMSE performance comparisons at different snapshots *L*. Where *M* = 8, *N* = 6, SNR is varied from 0 dB to 20 dB. We can observe that the RMSE performance is improved with the increase of snapshot *L*. When *L* increases, we get more samples to estimate the propagator matrix more accurately, and so the angle estimation performance is enhanced. 

**Figure 9:**
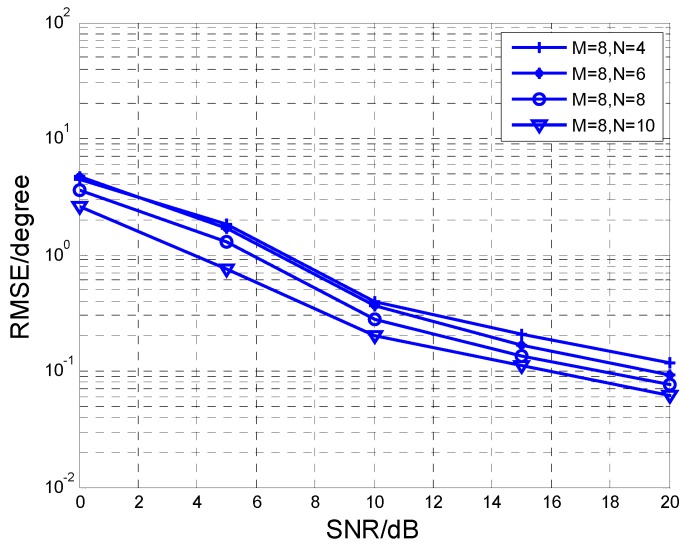
RMSE comparison at different *N* with *M* = 8.

**Figure 10:**
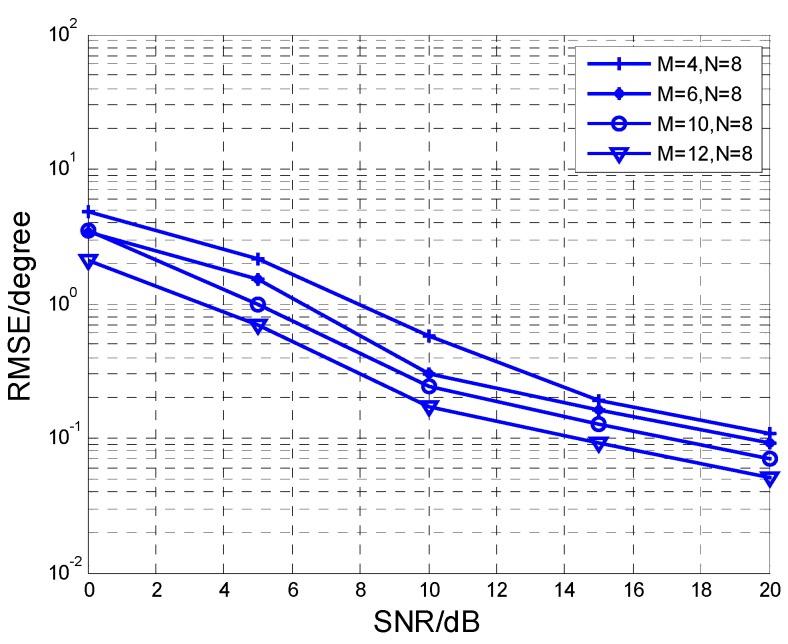
RMSE comparison at different *M* with *N* = 8.

Figure 9 and Figure 10 present RMSE versus different values of *M* or *N*, respectively. The snapshot *L* is fixed at 200. In addition, it is indicated that RMSE performance is improved when *M* or *N* increases. Multiple sensors enhance the aperture of the array as well as diversity gain. Therefore, it can improve the angle estimation performance.

**Figure 11:**
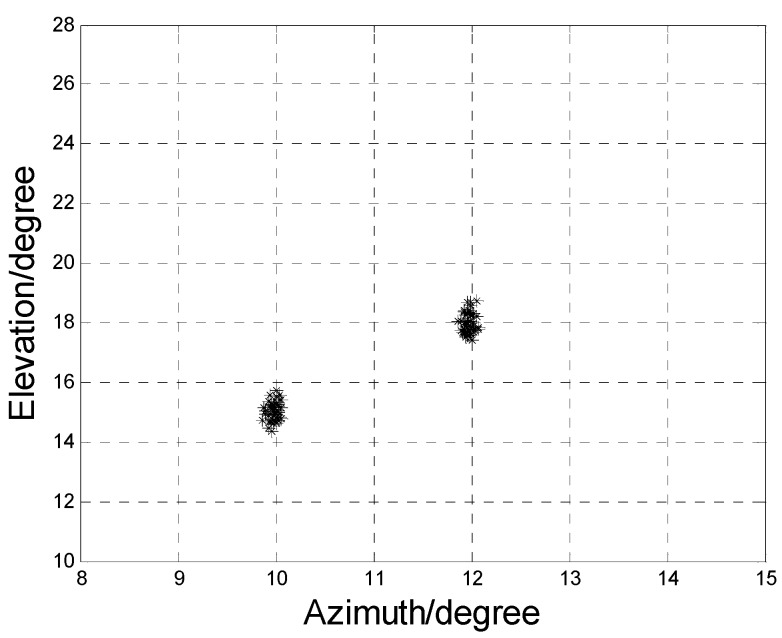
Scatter plot with closely spaced sources.

The estimation performance for two closely spaced sources is also investigated. Figure 11 depicts the scatter plot of 2D-DOA estimation results for two closely spaced sources. Where *M* = 8, *N* = 10, SNR = 10 dB, the snapshot *L* is 200. It is shown that our algorithm works well for the closely spaced sources.

## 6. Conclusions

We have presented a novel direction finding algorithm for uniform rectangular planar array. The characteristics of noncircular signal and Euler’s transformation are exploited to get the real-valued rectangular array data in a new way. The proposed algorithm can reduce the computational amount since it does not refer to plural operation and the eigenvalues’ decomposition of the covariance matrix. The theory analysis and simulation results verify that our algorithm is more suitable for real-time processing system in engineering.

## Abbreviations

2D-DOA	Two-dimensional direction of arrival
URA	Uniform rectangular planar array
PM	Propagator method
CRB	Crame–Rao bound
RMS	Real-valued multiplications
RMSE	Root mean square error
SNR	Signal-to-noise ratio
